# Current Insights into Potential Effects of Micro-Nanoplastics on Human Health by *in-vitro* Tests

**DOI:** 10.3389/ftox.2021.752140

**Published:** 2021-09-29

**Authors:** Marta Llorca, Marinella Farré

**Affiliations:** Department of Environmental Chemistry, IDAEA-CSIC, Barcelona, Spain

**Keywords:** nanoplastics, microplastics, human heath, toxicology, routes of exposure

## Abstract

Humans are exposed to micro and nanoplastics (MNPLs) through inhalation, ingestion and, to a lesser extent, dermal contact. In recent years, new insights indicate the potential of MNPLs to cause damages to human health. Particle toxicity can include oxidative stress, inflammatory lesions, and then increased internalization or translocation through tissues. On the other hand, plastic additives are used in plastic particles, once internalized, can release toxic substances. It is noteworthy that the potential effects of MNPLs encompass a wide range of polymers and chemical additives, showing various physicochemical and toxicological properties, and the size, shape and surface properties are other variables influencing their effects. In spite of the research carried out recently, MNPLs research is in its early stages, and further investigation is required. In this review article, the knowledge of human exposure routes and the recent results on the toxicological effects of MNPLs in human health are presented and discussed. Finally, the current limitations and the main gaps in the body of knowledge are summarised.

## Introduction

Fossil-based plastics have been one of the most impressive revolutions of the last century, contributing to improving the quality of life of humans. Nowadays, plastics are used in all of the industrial sectors because there is a wide variety of available versatile materials. They are inexpensive, strong, durable, lightweight, resistant to degradation, and present thermal and electrical insulation properties (Andrady and Neal, 2009). For that reason, the global production reached 368 million tonnes in 2019 (Plastics the Facts, 2019), and this trend is expected to continue in the coming years. However, as a consequence of incidental events and mismanaged wastes, plastic residues of different sizes can reach the environment and as a conseuence they are currently ubiquitous contaminants. While the problems caused by microplastics (MPLs) in the environment (Eerkes-Medrano et al., 2015; Horton et al., 2017), and in particular in marine ecosystems (Cole et al., 2011; Ivar Do Sul and Costa, 2014; Llorca et al., 2020a), is a well recognised problem, more recently there is increasing concern regarding the potential for human exposure to MPLs (Wright and Kelly, 2017). Due to the use of items made of plastic and the indirect impact of plastic pollution, humans are constantly exposed to micro- and nanoplastics (MNPLs) through the inhalation of fibres from clothing and fabrics, tyre dust from motor vehicles, ingestion of water with residues (from filters, pipes, and bottles), ingestion of contaminated foods, among many other routes of exposure (Prata et al., 2020).

MPLs have been defined as plastic pieces with a size below 5 mm (Besseling et al., 2013), including plastic particles at the nano range, known as nanoplastics (NPLs) (Bouwmeester et al., 2015). MPLs can have their origin in cosmetics and cleansing products, cloth fibres and vehicle tyre erosion (classified as primary MPLs), and the environmental fragmentation and erosion of plastic pieces and debris (classified as secondary MPLs) (Andrady, 2011). It is important to highlight that the toxicological impact of plastic pollution is partially influenced by the size and shape of the particles (Hwang et al., 2020), as is the case with other nanomaterials (Bouwmeester et al., 2015), as they have much smaller particles and greater ability to be internalised and translocated through tissues in living organisms. As a consequence, their potential toxicity also increases. Nothwithstanding, the smaller the particles, the greater their active surface area, hence they facilitate the leaching of plastic additives, which are chemical ingredients that are added to polymers to enhance their performance and characteristics, but they are not chemically bound to the polymeric chain. Therefore, they can easily leach from plastic particles being another route of exposure to these chemicals in addition to others. Moreover, some plastic additives have already been shown to have toxicological properties that are harmful to the environment and human health (Hermabessiere et al., 2017). On the other hand, the nature of the most used polymers facilitates that once in the environment MNPLs can adsorb and facilitate the transport of other environmental contaminants (Llorca et al., 2018; Llorca et al., 2020a) and pathogens to living organisms, which is known as the Trojan horse effect. This ability to adsorb and desorb other contaminants and pathogens is not only dependent on the polymeric composition of the particle, but is also size dependent because, again, the smaller the particles, the greater their active surface area.

However, to the best of our knowledge, to date the consequences of MNPL exposure on human health are not well understood. In addition, the extensive number of polymers and plastic additives that are involved, with variables such as the size and shape of the particles, and the current analytical limitations, should also be considered.

Under this frame this paper has been organised into the following sections: 1) main human routes of exposure; 2) pathways of particle toxicity; 3) toxicity of plastic additives on human health; 4) insights about Trojan horse effects on human health; and 5) future reaserch trends.

## Main Routes of Human Exposure

**FIGURE 1:**
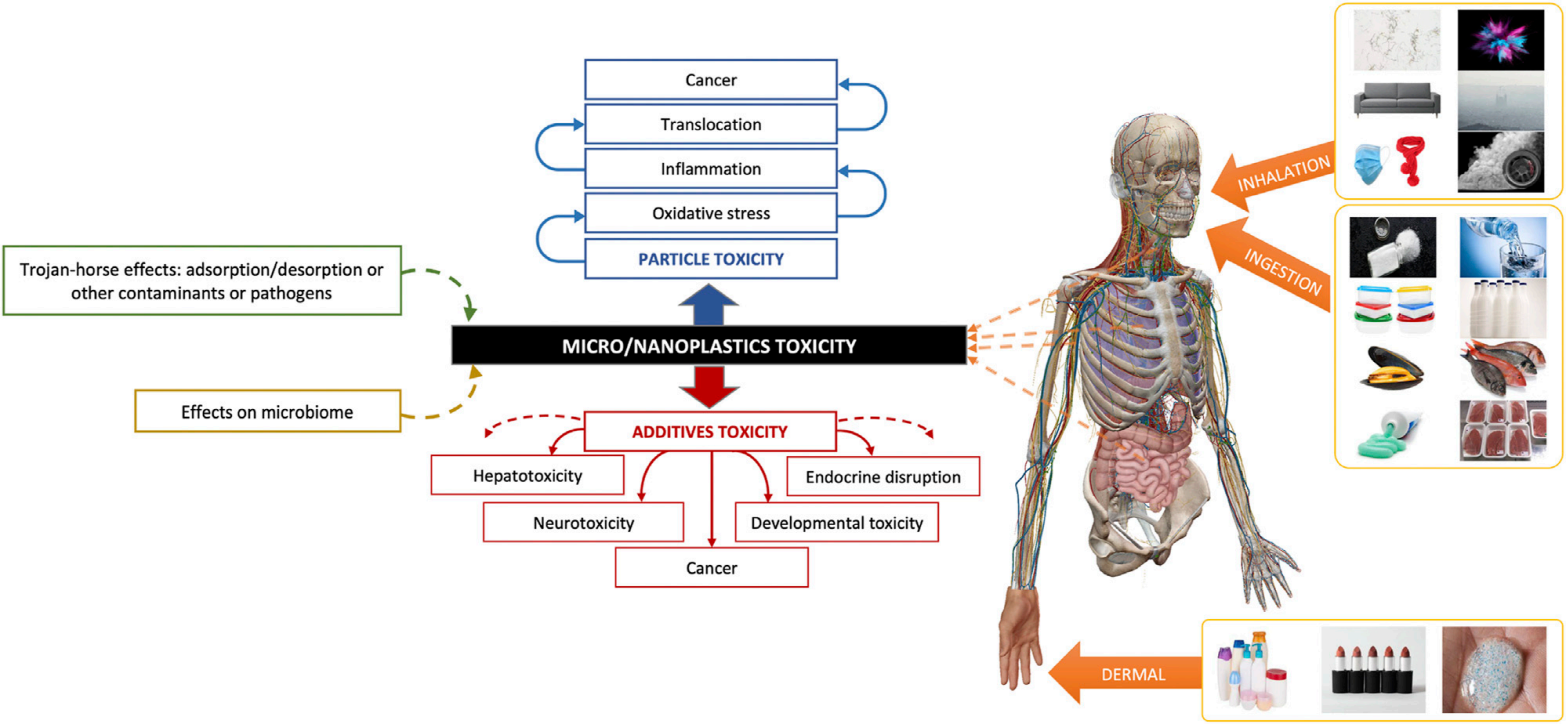
Summary of the human routes of exposure to MNPLs and their potential effects on health.

Recent studies indicate that MNPLs are present in most classes of consumer products. In foodstuffs, MNPLs can be present in animals which are contaminated through their environment or food chains, as happens with seafood (Santillo et al., 2017) for example, and also they can be contaminated during their production processes or packaging (Lau and Wong, 2000; Mason et al., 2018; Du et al., 2020). In the case of drinking water, MPL contamination can come from pipes, filters, or bottles (Oßmann, 2021). In addition to a portion of MNPLs that we can ingest incidentally, such as the MPLs that are present in some toothpaste formulas. Also, dust from plastics, car tyres, paints, and textile fibres are sources of MNPLs in the Earth’s atmosphere and can be inhaled or can undergo dermal interaction with humans. Moreover, MPLs are used in the formula of certain personal care products (PCPs) (Laborda et al., 2021), implying dermal interaction. Therefore, the main routes of human exposure to MNPLs are ingestion, inhalation, and dermal contact (Figure 1). However, the total contribution of MNPLs to exogenous particles exposure is still unknown, and due to their unique composition and properties, the human biological responses will differ from those of other exogenous materials.

Ingestion is considered as the major route of human exposure to MNPLs (Galloway, 2015), and the tissues of the human gastrointestinal tract (GIT) are considered among the most exposed. In particular, the Peyer’s patches of the ileum in the small intestine have potentially been defined as the main sites of uptake and translocation of anthropogenic particles. The Peyer’s patches are part of the gut-associated lymphoid tissues (Powell et al., 2010). The M cells of these patches can transport particles (0.1–10 μm) from the intestine to the mucosal lymphoid tissues, responsible for initiating immune responses to specific antigens that are encountered along all mucosal surfaces. The subepithelial area of the Peyer’s patches can store non-degradable particles, and consequently they disrupt the immunity processes (Campanale et al., 2020). For bigger particles, up to 130 μm diameter, persorption performed by GIT epithelial cells is another route of uptake but not of adsorption and is the cellular passage of large particles through the epithelial layer of the GIT. In addition to particle size, other factors that can influence the uptake and translocation of MNPLs are their chemical composition, shape, and hydrophobicity. Hydrophobic surfaces can be better transported through the mucus layer. Moreover, these characteristics also influence the adsorption of proteins and other biomolecules to the particle surface, known as corona formation (Lunov et al., 2011). Therefore, the surface charge of MNPLs can also influence the extent and pathway of translocation to other organs. Food and drinking water are key factors in the assessment of human MNPL exposure through ingestion, but most of the studies till now have been focused on fish and seafood, rather than on other foodstuffs.

MPLs in molluscs, such as mussels (Van Cauwenberghe and Janssen, 2014; Li et al., 2015; Smith et al., 2018), have been widely studied because they are living in intertidal areas, where the abundance of MPLs is expected to be higher due to the density of the polymers. For that reason, mussels are particularly exposed (Coppola et al., 2020). Moreover, the edible part of molluscs includes the stomach, intestine and digestive glandular, and they are consumed worldwide, thus potentially representing a source of MPLs in the human diet. As shown in Table 1, the concentrations of MPLs in molluscs present a high variability, for example, ranging from 0.05 to 3 MPLs items per g wet weight (ww) of edible tissue in mussels. This variability among molluscs is highly influenced by the habitat between different species and, in particular, the degree of contamination of the different sampled areas, in addition of the diversity of analytical methods used, including the diversity of the sample preparation approaches. Karlsson et al. (Karlsson et al., 2017) studied the accumulation of MPLs in mussels and the surrounding water and sediments, and their findings indicated that MPLs in mussels were approximately a thousand-fold higher, per unit weight or volume than in the surrounding sediment and water, showing the potential of bioaccumulation of filter-feeding organisms.

**TABLE 1:** Summary of studies assessing MNPLs in foodstuffs.

Foodstuffs	Polymers	Size of particles	No. of particles Average concentrations	Ref
Beer	—	Anthropogenic debris was found in each brand of beer; 99% were fibres	The average no. of particles found in beer was 4.05 particles/L with a range of 0–14.3 particles/L	Kosuth et al. (2018)
Chicken gizzards (used for human consumption)	—	16.45% of plastic particles found in the gizzard were smaller than 5 mm and 83.55% were > 5 mm	10.2 ± 13.8 microplastic particles per Gram	Huerta Lwanga et al. (2017)
Clam	PE, PET, and PA	Between 10 and 5,000 nm in diameter	The mean number of total encountered MPLs in all species ranged from 0.2 to 21.0 particles per g of soft tissue (wet weight) and from 3.7 to 17.7 particles per individual	Naji et al. (2018)
Amiantis umbonella and Amiantis purpuratus				
Oyster				
Pinctada radiata				
Mud snail				
Cerithidea cingulata				
Carnivorous snail				
*Thais* mutabilis				
Cockle	SBR, PS, PP, PET, PE, ABS, predominant in mussels	Minimum size 15 μm	Blue mussels and common cockles exhibited 0.76 ± 0.40 and 2.46 ± 1.16 MPLs/individual and between 0.15 ± 0.06 and 0.74 ± 0.35 MP L/g ww	Hermabessiere et al. (2019)
*Cerastoderma edule*	SBR copolymer and ABS in cockle			
Mussel	PE, PP Polyester (PES)			
*Mytilus edulis*	PE, PP Polyester (PES). PP, PA, PE, PS, PET, PVC, and PAN.			
Fish	PE, PP Polyester (PES). PP, PA, PE, PS, PET, PVC, and PAN.Fish contained higher percentages of fibres of PS, and PVC	>20 μm	MPLs were detected in gut and gills in 22–100% and 22–89% of total individuals	Su et al. (2019)
*Lateolabrax maculatus* Asian seabass			The size of MPLs in gills were smaller than those found in the gut. No MPLs were detected in the liver or muscle tissues	
Fish	Bivalves possessed higher percentages of fragments, films, and particles of PET.	Bivalves contained higher percentages of shorter fibres in MPLs (0.1–1.0 mm) than fish, but fish contained higher percentages of longer MPLs (>1.0 mm) than bivalves		Fang et al. (2019b)
Konosirus punctatus	PE, polyester			
*Acanthopagrus* latus	PE, polyester PEVA and HDPE			
Perna viridis	PE, polyester PEVA and HDPE PEVA and HDPE PP and PET			
Meretrix	PEVA and HDPE. PP and PET			
Konosirus punctatus	PE, polyester PEVA and HDPE			
*Acanthopagrus* latus	The most abundant polymer was PE in both edible (30.4%) and inedible tissues (22.4%), followed by PP (17% in edible and 18% in inedible tissues).The most abundant polymer was PE in both edible (30.4%) and inedible tissues (22.4%), followed by PP (17% in edible and 18% in inedible tissues). The most abundant polymer was PE in both edible (30.4%) and inedible tissues (22.4%), followed by PP (17% in edible and 18% in inedible tissues). Polyester or PET, and Poly (ether-urethane)			
*Mytilus edulis*	The most abundant polymer was PE in both edible (30.4%) and inedible tissues (22.4%), followed by PP (17% in edible and 18% in inedible tissues). The most abundant polymer was PE in both edible (30.4%) and inedible tissues (22.4%), followed by PP (17% in edible and 18% in inedible tissues). The most abundant polymer was PE in both edible (30.4%) and inedible tissues (22.4%), followed by PP (17% in edible and 18% in inedible tissues). Polyester or PET, and Poly (ether-urethane). PEVA and HDPE. PP and PET.			
Meretrix	The most abundant polymer was PE in both edible (30.4%) and inedible tissues (22.4%), followed by PP (17% in edible and 18% in inedible tissues). The most abundant polymer was PE in both edible (30.4%) and inedible tissues (22.4%), followed by PP (17% in edible and 18% in inedible tissues). The most abundant polymer was PE in both edible (30.4%) and inedible tissues (22.4%), followed by PP (17% in edible and 18% in inedible tissues). Polyester or PET, and Poly (ether-urethane)			
Fish; 3 species	The most abundant polymer was PE in both edible (30.4%) and inedible tissues (22.4%), followed by PP (17% in edible and 18% in inedible tissues). The most abundant polymer was PE in both edible (30.4%) and inedible tissues (22.4%), followed by PP (17% in edible and 18% in inedible tissues). The most abundant polymer was PE in both edible (30.4%) and inedible tissues (22.4%), followed by PP (17% in edible and 18% in inedible tissues). Polyester or PET, and Poly (ether-urethane) PEVA and HDPE PP and PET. PEVA and HDPE PP and PET. The most abundant polymer was PE in both edible (30.4%) and inedible tissues (22.4%), followed by PP (17% in edible and 18% in inedible tissues).		0.4 ± 0.7 MPLs items/g; 0.7 ± 1.3 MPLs items/g; and 0.6 ± 0.8 MPLs items/g in the dorsal muscle of *D. labrax, T. trachurus* *and S. colias*, respectively	Barboza et al. (2020)
*Dicentrachus labrax*			The total mean (±SD) of the number of MPLs in the dorsal muscle was 0.054 ± 0.099 items/g	
*Trachurus*				
*Scomber colias*				
Fish	The most abundant polymer was PE in both edible (30.4%) and inedible tissues (22.4%), followed by PP (17% in edible and 18% in inedible tissues). The most abundant polymer was PE in both edible (30.4%) and inedible tissues (22.4%), followed by PP (17% in edible and 18% in inedible tissues). Polyester or PET, and Poly (ether-urethane). Polyester or PET, and Poly (ether-urethane) Polyester or PET, and Poly (ether-urethane)		Average concentrations in edible parts 0.2 ± 0.3 particles/g tissue.	Zitouni et al. (2020)
*Serranus scriba* - lettered perch				
Fish; canned sardines and sprats originating from 13 countries	The most abundant polymer was PE in both edible (30.4%) and inedible tissues (22.4%), followed by PP (17% in edible and 18% in inedible tissues). Polyester or PET, and Poly (ether-urethane). Polyester or PET, and Poly (ether-urethane). Polyester or PET, and Poly (ether-urethane). Polyester or PET, and Poly (ether-urethane). Polyester or PET, and Poly (ether-urethane)Polyester or PET, and Poly (ether-urethane). PU, PVCA, PVC, PES, PVC/Acrylic alloy, PET, PVK, PEVA.	>149 μm	1 and 3 plastic particles	Karami et al. (2018)
Fish, crab, and prawn	The most abundant polymer was PE in both edible (30.4%) and inedible tissues (22.4%), followed by PP (17% in edible and 18% in inedible tissues). Polyester or PET, and Poly (ether-urethane) olyester or PET, and Poly (ether-urethane) Polyester or PET, and Poly (ether-urethane)		*Penaeus* semisulcatus and *Epinephelus coioides* displayed the highest (mean 0.360 items/g muscle) and lowest (mean 0.158 items/g muscle)	Akhbarizadeh et al. (2019)
Fish pelagic species: Rastrelliger kanagurta, Megalaspis cordyla	The most abundant polymer was PE in both edible (30.4%) and inedible tissues (22.4%), followed by PP (17% in edible and 18% in inedible tissues). Polyester or PET, and Poly (ether-urethane). Polyester or PET, and Poly (ether-urethane). Polyester or PET, and Poly (ether-urethane) Polyester or PET, and Poly (ether-urethane) Polyester or PET, and Poly (ether-urethane) Polyester or PET, and Poly (ether-urethane) PU, PVCA, PVC, PES, PVC/Acrylic alloy, PET, PVK, PEVA. Polyester or PET, and Poly (ether-urethane) Polyester or PET, and Poly (ether-urethane) Polyester or PET, and Poly (ether-urethane) PU, PVCA, PVC, PES, PVC/Acrylic alloy, PET, PVK, PEVA. PU, PVCA, PVC, PES, PVC/Acrylic alloy, PET, PVK, PEVA. PU, PVCA, PVC, PES, PVC/Acrylic alloy, PET, PVK, PEVA. Eighteen types of polymers detectedPU, PVCA, PVC, PES, PVC/Acrylic alloy, PET, PVK, PEVA. Eighteen types of polymers detected PVC and rayon being the most abundant types	<100 μm	0.07 ± 0.26 items per fish in edible tissues	Daniel et al. (2020)
*Sardinella* longiceps				
*Sardinella gibbosa*				
Stolephorus indicus, Dussumieria acuta				
Thryssa dussumieri				
*Sphyraena obtusata* and				
Anodontostoma chacunda				
Fruit	Polyester or PET, and Poly (ether-urethane) Polyester or PET, and Poly (ether-urethane) Polyester or PET, and Poly (ether-urethane) PU, PVCA, PVC, PES, PVC/Acrylic alloy, PET, PVK, PEVAPU, PVCA, PVC, PES, PVC/Acrylic alloy, PET, PVK, PEVA. PU, PVCA, PVC, PES, PVC/Acrylic alloy, PET, PVK, PEVA. Eighteen types of polymers detected. PU, PVCA, PVC, PES, PVC/Acrylic alloy, PET, PVK, PEVA. Eighteen types of polymers detected. PVC and rayon being the most abundant types -PU, PVCA, PVC, PES, PVC/Acrylic alloy, PET, PVK, PEVA. PU, PVCA, PVC, PES, PVC/Acrylic alloy, PET, PVK, PEVA. Eighteen types of polymers detected. PU, PVCA, PVC, PES, PVC/Acrylic alloy, PET, PVK, PEVA. Eighteen types of polymers detected. PVC and rayon being the most abundant types PU, PVCA, PVC, PES, PVC/Acrylic alloy, PET, PVK, PEVA.	In M. Domestica median size 2.17 μm	*MNPs particles in M. Domestica* 195,500, in P. Communis 189,550, in *B. oleracea* *italica* 126,150, in *L. Sativa* 50,550, and in *D. Carota* 101,950	(Oliveri Conti et al., 2020)
*Malus domestica* - apple		In *P. communis* median size 1.99 μm		
*Pyrus communis* - pear		In *B. oleracea* italica Domestica median size 2.10 μm		
Vegetables		In *L. Sativa* median size 2.52 μm		
*B. oleracea italic*- broccoli		In *D. Carota* median size 1.51 μm		
*Lactuca sativa* - lettuce		20 μm		
Daucus carota - carrots				
*Solanum tuberosum* - potato		20 μm. MPL length ranged from 30 to 2000 μm with a median length of 200 μm		
Mussel species	PU, PVCA, PVC, PES, PVC/Acrylic alloy, PET, PVK, PEVA.PU, PVCA, PVC, PES, PVC/Acrylic alloy, PET, PVK, PEVA. Eighteen types of polymers detected PU, PVCA, PVC, PES, PVC/Acrylic alloy, PET, PVK, PEVA. Eighteen types of polymers detected. PVC and rayon being the most abundant types. PU, PVCA, PVC, PES, PVC/Acrylic alloy, PET, PVK, PEVA. PU, PVCA, PVC, PES, PVC/Acrylic alloy, PET, PVK, PEVA.	20 μm. MPL length ranged from 30 to 2000 μm with a median length of 200 μm 61.02 and 77.42% of the particles belonged to the size group of <100 μm in *M. meretrix* and *P. viridis*, respectively	In these mussels, the mean load of observed particles was 3 ± 0.9 particles/g ww, equivalent to 3.2 ± 0.52 particles per mussel. In M. modiolus the concentration of MPLs were substantially heavier, 42.91 ± 2.111 g	Catarino et al. (2018)
*Mytilus edulis*				
*Mytilus* spp. and the subtidal				
*Modiolus*				
Mussel	PP, PA, PAN, PE, PEVA, CP. PP, PA, PAN, PE, PEVA, CP. Several but PE being the predominant	61.02 and 77.42% of the particles belonged to the size group of <100 μm in *M. meretrix* and *P. viridis*, respectivelyMPL length ranged from 30 to 2000 μm with a median length of 200 μm	Average concentration 0.2 ± 0.3 particle/g tissue	Van Cauwenberghe et al. (2015)
*Mytilus edulis*				
Mussel	PP, PA, PAN, PE, PEVA, CP. PP, PA, PAN, PE, PEVA, CP. Several but PE being the predominant. PP, PA, PAN, PE, PEVA, CP. Several but PE being the predominant Several but PE being the predominant. PE	MPL length ranged from 30 to 2000 μm with a median length of 200 μm. Microfibre was the most predominant shape with diameters between 7 and 5,000 μm	Average amount: 37,000 (*σ* = 25,000) microplastics kg/dw	Karlsson et al. (2017)
*Mytilus edulis*				
Mussel	PP, PA, PAN, PE, PEVA, CP. PP, PA, PAN, PE, PEVA, CP Several but PE being the predominant. PP, PA, PAN, PE, PEVA, CP. Several but PE being the predominant. Several but PE being the predominant. PE. Several but PE being the predominant. PE	Microfibre was the most predominant shape with diameters between 7 and 5,000 μm Microfibre was the most predominant shape with diameters between 7 and 5,000 μm. Particle size varied from 36 to 4,439 μm, being fibers the most abundant shape (50%) followed by films (22%). 150–6,000 μm	Average abundance	Dowarah et al. (2020)
*Perna viridis*			0.18 ± 0.04 g tissue ww, 1.84 ± 0.61 g tissue ww and 1.76 ± 0.48 g tissue ww; and the number of MPLs per bivalve is 0.50 ± 0.11, 1.75 ± 0.35, and 4.80 ± 1.39, respectively, for the 3 locations sampled: Ariyankuppam, Panithittu, and Chunnambar	
*Meretrix*				
Mussel	PE. CP, PE and PET	Microfibre was the most predominant shape with diameters between 7 and 5,000 μm. Particle size varied from 36 to 4,439 μm, being fibers the most abundant shape (50%) followed by films (22%). 150–6,000 μm. Particle size varied from 36 to 4,439 μm, being fibers the most abundant shape (50%) followed by films (22%). 150–6,000 μm	Average ranging from 0.5 to 3.3 items/individual	Ding et al. (2021)
*Mytilus galloprovincialis,* oyster	PP, PA, PAN, PE, PEVA, CP. Several but PE being the predominant		Detected in the 80% of the samples over four seasons	
*Crassostrea gigas*, clam	CP, PE and PET. CP, PE and PET. PET, polyester, and PA, cellulose acetate and CP			
*Ruditapes philippinarum*	CP, PE and PET. CP, PE and PET. PET, polyester, and PA, cellulose acetate and CP. Several but PE being the predominant. PE. CP, PE and PET, polyester, and PA, cellulose acetate and CP PET, polyester, and PA, cellulose acetate and CPXPS			
Scallop	CP, PE and PET, polyester, and PA, cellulose acetate and CP			
*Chlamys farreri*	Several but PE being the predominant PE. CP, PE and PET. PET, polyester, and PA, cellulose acetate and CP. PET, polyester, and PA, cellulose acetate and CP XPS PE CP, PE and PET			
Mussel	Several but PE being the predominant PE CP, PE and PET PET, polyester, and PA, cellulose acetate and CP PET, polyester, and PA, cellulose acetate and CP XPS PE CP, PE and PET PE CP, PE and PET, polyester, and PA, cellulose acetate and CP XPS	Particle size varied from 36 to 4,439 μm, being fibers the most abundant shape (50%) followed by films (22%) 150–6,000 μm. 150–6,000 μm. Mean length ±SD (cm)	MPLs concentrations ranged from 0.54 to 3.0 items/g without significant differences among the sites	Marques et al. (2021)
*Mytilus* spp.				
Mussel	PE CP, PE and PET PET, polyester, and PA, cellulose acetate and CP XPS PET, polyester, and PA, cellulose acetate and CP XPS polyesters, PET, PA, PE, PS polyesters, PET, PA, PE, PS PE, PP, PS	150–6,000 μm Mean length ±SD (cm) Oysters 8.30 ± 0.45. Mussels 1.24 ± 0.14	0.20 ± 0.24 items/g ww; 0.40 ± 0.47 items/individual	Nalbone et al. (2021)
*Mytilus galloprovincialis*				
Mussel (processed)	PET, polyester, and PA, cellulose acetate and CP XPS polyesters, PET, PA, PE, PS. polyesters, PET, PA, PE, PS PE, PP, PS. PET, polyester, and PA, cellulose acetate and CP XPS. polyesters, PET, PA, PE, PS polyesters, PET, PA, PE, PS. PE, PP, PS. polyesters, PET, PA, PE, PS. polyesters, PET, PA, PE, PS. PE, PP, PS. PP, PE, PA and cellulose	Oysters 8.30 ± 0.45. Mussels 1.24 ± 0.14. Fibres were the most common shape (60.67%), and the most common size was <1,500 μm	0.9 ± 0.10 items/g ww; 0.17 ± 0.19 items/individual	Nalbone et al. (2021)
*Mytilus galloprovincialis*				
Mussel and oyster	PET, polyester, and PA, cellulose acetate and CP XPS. polyesters, PET, PA, PE, PS. polyesters, PET, PA, PE, PS. PE, PP, PS. polyesters, PET, PA, PE, PS polyesters, PET, PA, PE, PS PE, PP, PS PP, PE, PA and cellulose polyesters, PET, PA, PE, PS polyesters, PET, PA, PE, PS PE, PP, PS PP, PE, PA and cellulose PP, PE, PA and cellulose Polyester, PVC, PA, PE	Oysters 8.30 ± 0.45	Average amounts: *Crassostrea gigas*	Bonello et al. (2018)
*Mytilus galloprovincialis*		Mussels 1.24 ± 0.14	0.11 MPLs/g	
*Crassostrea gigas*		Over 99% of suspected MPLs encountered were microfibres with an average length of 1.33 ± 0.04 mm (range = 0.11–7.84 mm) 4.0 to 18.7 MP-XPS/kg of packaged meat	*Mytilus galloprovincialis* 0.05 MP L/g	
Oyster	polyesters, PET, PA, PE, PS polyesters, PET, PA, PE, PS PE, PP, PS PP, PE, PA and cellulose PP, PE, PA and cellulose Polyester, PVC, PA, PE, PE, PP, PPS, PS. PET, PPT, epoxy resin, Rayon, PET, PE, PS,Polyester, PAA, PMPS, PI	Over 99% of suspected MPLs encountered were microfibres with an average length of 1.33 ± 0.04 mm (range = 0.11–7.84 mm) 4.0 to 18.7 MP-XPS/kg of packaged meat 4–2,100 μm in Italian salt	Average abundance of MPLs in oyster was 0.62 items/g ww or 2.93 items/individual	Teng et al. (2019)
*Crassostrea gigas*				
*Crassostrea angulate, Crassostrea hongkongensis, Crassostrea sikamea*				
Pacific razor clam	PE, PP, PPS, PS. PET, PPT, epoxy resin, Rayon, PET, PE, PS,Polyester, PAA, PMPS, PI, PPT, epoxy resin, Rayon, PET, PE, PS,Polyester, PAA, PMPS, PI, PET, PP, PE or polyoleofins	4–2,100 μm in Italian salt 15–4,628 μm in Croatian salt	Average suspected MPLs 6.75 ± 0.60 MP L/g ww	Baechler et al. (2020)
*Siliqua patula*				
Poultry meat (packed)	PPT, epoxy resin, Rayon, PET, PE, PS,Polyester, PAA, PMPS, PI	80% of the extracted fibres and the fragments were smaller than 2000 and 500 μm, respectively	130 and 250 μm	Kedzierski et al. (2020)
Sea salt	PP, PET, PS, and PP	40–170 µm	The average no. of particles found in each brand of salt was 212 particles/kg with a range of 46.7–806 particles/kg	Kosuth et al. (2018)
Sea salt	PP, PET, PS, and PP. PP PET, PS, and PP	MPLs that measured less than 100 μm formed major part of the salts, accounting for 60% of the MPLs among the total pollutants	1.57–8.23 MPLs/g Italian salt	Renzi and Blašković, (2018)
		The majority were fibres (98.3%) 0.1–5 mm	27.13–31.68 MPLs/g in Croatian salt	
Sea salt	PPT, epoxy resin	42% of the samples containing MPLs fragments>20 μm	particles ranged from 103 ± 39 to 56 ± 49 MPLs/kg of salt	Seth and Shriwastav, (2018)
Sea salt		95% of the samples containing MPLs. Fragments > fibres > spheres >50 μm	Approx. 1,000 MPLs/kg	Cho et al. (2019)
Sea salt		100% of the samples containing MPLs. Fibres > fragments		Selvam et al. (2020)
Water (Tap-water)		100% of the samples containing MPLs. Fibres > fragments	Mean value 5.45 particles/L	Kosuth et al. (2018)
. Water (Tap-water)		Small MPLs (–50–500 μm) and very small (1–50 μm) fragments were found in every type of water. Almost 80% of all MNPLs found had a particle size between 5 and 20 μm	0–0.0007 MP L/L	Mintenig et al. (2019)
Water (Tap-water)			0–1,247 MPLs/L	Tong et al. (2020)
Water (Tap-water)		Fragments were the most common morphology (66%)	5 ± 2 to 91 ± 14	Shruti et al. (2020b)
Water (Tap-water)		>100 um	0.3 to 1.6 MPLs/L	Zhang et al. (2020b)
Water (bottled)		The most detected MPLs were fragments (93%) and fibre (7%)	Average MPL content was 118 ± 88 particles/L in returnable, but only 14 ± 14 particles/L in single-use plastic bottles	Schymanski et al. (2018)
Water (bottled)			10.4 MPLs/L	Mason et al. (2018)
			Including smaller particles (6.5–100 um), an average of 325 MPLs/L of bottled water. MPLs contamination range of 0 to over 10,000 MPLs/L with 95% of particles being between 6.5 and 100 um in size	
Water (bottled)			Average concentration was approximately 8.5 ± 10.2 particles/L	Makhdoumi et al. (2021)

acrylonitrile-butadiene-styrene (ABS); cellophane (CP); high density polyethylene (HDPE); polyamide (PA); polyacrylic acid (PAA); polyacrylonitrile (PAN); polyethylene (PE); poly (p-phenylene ether sulfone) (PES); polyethylene terephthalate (PET); polyethylene-vinyl-acetate (PEVA); polyimide (PI); polymethyl pentene (PMPS); Polyester; polypropylene (PP); polyphenylene sulfide (PPS); polystyrene (PS); polyester urethane (PU); polyvinyl chloride (PVC); vinyl chloride/vinyl acetate copolymer (PVCA); poly (N-vinyl carbazole) (PVK); styrene butadiene rubber copolymer (SBR); extruded polystyrene (XPS).

As can be seen in Table 1, the prevalent morphotypes were microfibres. Regarding polymers, the most detected were polyethylene (PE), polypropylene (PP), polyethylene terephthalate (PET), and the polyamide group (PA).

On the other hand, despite the number of studies on MPLs in the GIT of fishes (Schirinzi et al., 2020; Abidli et al., 2021; Hadibarata et al., 2021; Ghosh et al., 2021; Li et al., 2021; Zhang et al., 2021), only a reduced number of studies considered the edible parts. Daniel et al. (2020) (Daniel et al., 2020) recently carried out a study in which they compared the MPLs in edible and non-edible parts of pelagic fish for human consumption, showing that MPLs in the GIT of fishes was much higher than in edible parts, such as muscle. In another study, Barbosa et al. (2020) (Barboza et al., 2020) studied the distribution of MPLs in the GIT, gills and dorsal muscle of 3 fish species, *Dicentrachus labrax, Trachurus, Scomber colias*, and 49% had MPLs, while 32% had MPLs in dorsal muscle, with a total mean (±SD) of 0.054 ± 0.099 MPLs items/g. The results again showed higher results in the GIT and gills than in the edible parts of the 3 studied species. 1.3 ± 2.5; 1.0 ± 1.9; 1.2 ± 1.6 MPLs per individual in the GIT, 0.8 ± 1.4; 0.7 ± 1.4; 0.7 ± 1.0 MPLs per individual in the gills, and 0.4 ± 0.7; 0.7 ± 1.3; 0.6 ± 0.8 MPLs per g, in the dorsal muscle of *D. labrax*, *T. trachurus*, and *S. colias*, respectively. In this regard, it should be pointed out that only the smallest particles can be internalised and translocated, while to date due to technical reasons, most of the studies are not considering the evaluation of NPLs,. Therefore, there is an important gap in the knowledge about NPL contamination in the edible parts of fishes.

Another source of MNPL contamination can be packaging. Nevertheless, there are scant studies that have considered the potential of MNPL transfer from plastic cans (holding drinks or food) and plastic films. Recently, Akhbarizedeh et al. (Akhbarizadeh et al., 2020a) assessed the abundance and composition of MPLs in canned fish. This study showed that 80% of samples had at least one MPL item, with fibres being the most abundant example. PET was present in 32% of the samples, which was the most commonly found polymer (Akhbarizadeh et al., 2020a). In another study (Karami et al., 2018), MPL contamination was studied in sardine (Sardina *pilchardus*) and sprat (*Sprattus*). In this case, 20 brands of canned sardines and sprats that had been collected in 13 countries were examined. MPLs were confirmed in only four brands, within which, between 1 and 3 MPL particles were found, and polypropylene (PP) and polyethylene terephthalate (PET) were the identified polymers.

In addition to seafood and fish, drinking water is a significant potential source of MNPLs in the human diet. As shown in Table 1, several studies reported the presence of MNPLs in potable tap water. In these studies, both fibres and fragments had been identified. Despite the fact that PVC is one of the most used polymers in pipes was marginally detected in tap water, while polyethylene (PE), Polystyrene (PS), PET and fibres of polyester were those that were more frequently confirmed. In most of the studies, MNPLs in tap water were present in frequencies superior to 90%. However, the concentration of the particles was very variable between the different studies. In this case, the results were highly dependent on the different analytical approaches used, ranging from optical microscopy, scanning electron microscopy (SEM), techniques based on Fourier Transform Infrared (FTIR) spectrometry, and Raman spectrometry, and their capacity to measure the range of the smallest particles. Harmonised methods are urgently needed for drinking water and other food matrices (Oßmann, 2021). Moreover, in the case of drinking water it is particularly relevant to measure the range from NPLs to a few μm, as well as the combination with other analytical techniques that are able to provide polymer concentrations per litre of water, such as liquid chromatography coupled to high-resolution mass spectrometry (LC-HRMS) (Schirinzi et al., 2019) or gas chromatography pyrolysis mass spectrometry (GC-Pyr-MS) because concentrations in terms of the mass of polymer in water can differ in the counting particles of each polymer, and other techniques are not able to provide an assessment of NPLs. Nevertheless, from the toxicological perspective, especially the smallest MNPLs (<1.5 μm) might be toxicologically relevant, in agreement with the European Food Safety Authority (EFSA) (Efsa, 2016).

To carry out the toxicological assessment, often only particle numbers and sizes are used, and only sometimes also concentrations in mass per litre are considered. Recently, the World Health Organization (WHO), in its report on MPs in drinking water (Who, 2019), revisited this issue.

As happens in tap water, high variability was found when comparing different studies on bottled water (Akhbarizadeh et al., 2020b). The most common polymer that was found in bottled water was PET, which is generally used for bottle production and also PP, which is used for the caps. Therefore, these data suggest that the contamination is at least partially coming from the packaging and/or the bottling process itself.

Another studied foodstuff matrix was sea salt, but as happens with water, the comparison of the different studies cannot be made, in particular, because the different studies were carried out using different techniques (optical microscopy, FTIR, GC-Pyr-MS), and some of these techniques, such as optical microscopy, only provide information on bigger particles.

A minor number of studies considered other food matrices such as soft drinks (Shruti et al., 2020a). Thus, very few studies estimated the intake of MPLs through the diet. Focussing on the diet in the United States, Cox et al. (2019) (Cox et al., 2019) estimated the intake of MPLs. Evaluating approximately 15% of American’s caloric intake, MNPL consumption ranges were from 39,000 to 52,000 particles/person per year depending on age and sex. Additionally, individuals who drink only bottled water may be ingesting an additional 90,000 MNPLs annually, compared to 4,000 MNPLs for those who consume only tap water. Notwithstanding, food preparation and cooking can influence the average MNPL concentrations in meals (Rist et al., 2019). Thus, the comparison between raw foods and final meals is required to assess human exposure to MPLs through the diet.

### Inhalation

MNPL inhalation is considered to be one of the main routes of human exposure. Once inhaled, MNPLs reach the respiratory epithelium and, according to the lessons learned from particulate matter and nanomaterials investigations, it is expected that they may translocate via diffusion, direct cellular penetration, or active cellular uptake through endocytic and phagocytic processes. In the alveoli, phagocytosis is the main pathway for the particles with sizes of between 1 and 3 μm, while the smallest particles could be passively transported via diffusion across membrane pores (Wright and Kelly, 2017). Nonetheless, the number of studies assessing MNPLs in indoor and outdoor environments continues to be scant (Huang et al., 2020). Most of the studies that have been carried out to date were based on the use of μ-FTIR, which limits the assessment of NPLs. These approaches are, in general, composed of sample collection using passive or active samplers with quartz fibre filters (pore size: 2 μm, diameter: 46.2 mm). In most of the sample pre-treatment procedures, after collecting the total suspended particulate, the samples are digested to remove the organic materials. The most widely used digestion approach is the use of a H_2_O_2_ solution of 30% (v/v), sometimes with heating to speed up the process. Similar approaches are also well used in the assessment of atmospheric fallout but, in general, they are using passive collectors with different types of filters such as glass fibre filters (Dris et al., 2015), quartz fibre filters (Dris et al., 2016), nitrocellulose filters (Zhou et al., 2017), and PTFE filters (Allen et al., 2019).

As shown in Table 2, fibres are generally the most common MPLs that are identified in outdoor atmospheres. The analytical method that is employed can limit the range of particles that can be assessed. Nowadays, there is a continued existence of a critical gap of knowledge about the minor-sized particle fraction, which can be inhaled. On the other hand, relatively few research articles have considered indoor environments. In order to assess human exposure to indoor airborne MPLs, a breathing thermal manikin was used by Vianello et al. (Vianello et al., 2019) and those authors investigated MPLs down to 11 µm particle size. The manikin was used to investigate 3 different habited apartments, and the concentrations were found between 1.7 and 16.2 particles m^−3^, but only 4% were MPLs, with polyester being the predominant polymer in 81%, followed by PE in 5%, and PA in 3%. Another relevant result of this study was that the MPLs were typically of a smaller size than the non-synthetic particles. Zhang et al. (2020a) (Zhang et al., 2020a) studied MPL fallout in different indoor environments, and they established that dormitories were the rooms with a higher abundance of MPLs (average 9,900 MPLs/m^2^/day). In addition, airflow turbulence by the use of an air conditioner increased the resuspension of MPLs. In another study, the presence of MPLs in indoor dust was investigated in the city of Surabaya (Indonesia). The deposition was assessed in an apartment, an office, and a school (Bahrina et al., 2020). The greatest concentration was found in the office with an average of 1,186.36 particles/m^2^, and most of the MPLs were fibres. PET, polyester, and cellophane (CP) were the main polymers detected. Recently, in another study, thirty-two airborne indoor deposited dust samples were studied in homes in Sydney (Australia) (Soltani et al., 2021). MPL fibre deposition rate ranged from 22 to 6,169 fibres/m^2^/day, and most of the deposited dust were fibres (99%). The majority were natural fibres (42%), 18% were transformed natural fibres, and 39% were MPLs. PE, polyester, PA, and PS were found in higher abundance in homes with carpets. While when the carpet was absent, the polyvinyl fibres were predominant. Mean inhaled MPL weight was estimated to be 0.2 ± 0.07 mg/kg-body weight (BW)/year and 12,891 ± 4,472 fibres/year. It is noteworthy that the greatest inhalation intake rates were for the age group below 0.5-years, at 0.31 mg/kg-BW/year. As can be seen in these studies, there is a common trend indicating that fibres have predominant shapes. However, the rate of deposition and the rates of inhalation can be extremely different according to many factors, such as climate, the use of carpets or an air conditioner, the use of vacuum cleaners, types of textiles, number of habitants in the same place (Pratiwi et al., 2020), among many others, can affect the final rates.

**TABLE 2:** Summary of studies assessing MNPLs in airborne particulate and atmospheric deposition.

**Place**	**Size range**	**Abundance**	**Shape**	**Polymers**	**Ref**
**Outdoor**					
China (21 transects from the Pearl River Estuary to the South China Sea and then to the East Indian Ocean	Pearl River estuary	Pearl River estuary	Pearl River estuary	PET, PP, PA, PEP, PAN-AA, PR, PEVA	Wang et al. (2020b)
	288.2–1,117.62	4.2 ± 2.5 MPLs/100 m^3^	Fibres		
	South China Sea	South China Sea	South China Sea		
	58.591–988.37	0.8 ± 1.3 MPLs/100 m^3^	Fibres 80%, fragments 20%		
	East Indian ocean	East Indian ocean	East Indian ocean		
	286.10–1861.78	0.4 ± 0.6 MPLs/100 m^3^	Fibres 75%, fragments 25%		
China (Yantai)	50–1,000	Range: 130–624 MPLs/m^2^/d	Fibres, foam, film, fragments	PET, PVC, PE, PS	Zhou et al. (2017)
France (urban area of Paris)	50–3,200	Range	Fibres	RY, PET, PU	Dris et al. (2016)
		2–355 MPLs/m^2^/d; average			
		110 ± 96 MPLs/m^2^/d			
France (suburban area of Paris)	50–3,200	Mean	Fibres	RY, PET, PU	Dris et al. (2016)
		53 ± 38 MPLs/m^2^/d			
France (Paris)	59-1650μ	Range	Fibres	--	Dris et al. (2017)
		0.3–1.5 MPLs/m^3^ average 0.9 MPLs/m^3^			
France (Pyrenees; mountain range)	50–700	Average	Fibres	PS, PE, PP, PVC, PET	Allen et al. (2019)
		365 ± 69 MPLs/m^2^/d			
Iran (Asaluyeh - County-urban area)	2–5,000	Average	Fibres	--	Abbasi et al. (2019)
		0.63 MPLs/m^3^			
New Zealand (Christchurch- Suburban area)			Fibres		Knobloch et al. (2021)
West Pacific Ocean	20–2000	Coastal area (0.13 ± 0.24 MPLs/m^3^)	Fibres, fragment, and granule quantitatively constituted 60, 31, and 8% of all MPLs, respectively	PET, EP, PE-PP, PS, PE, PVC, Phe, ALK, PMA, PA, PVA, PAN, PP	Liu et al. (2019b)
		Pelagic area (0.01 ± 0.01 MPLs/m^3^)			
		Daytime (0.45 ± 0.46 MPLs/m^3^) was twice the amount collected at night (0.22 ± 0.19 MPLs/m^3^), on average			
**Indoor**					
France (Paris)	50–5,000	Average 190–670	Fibres	---	Dris et al. (2017)
		MPLs/mg			
China	50–2000	Range of fibres: 17–620 fibres/mg, mean of fibres: 342 fibres/mg; range of granules: 6–184 particles/mg	Fibres, granule	PET, PAN, PA, PE, PP, PU, PEI, acrylic, alkyd, cellulose, rayon	Liu et al. (2019c)
France (Paris)	50–3,250	Range	Fibres	RY, PA, PE, PP	Dris et al. (2016)
		0.4–59.4 particles/m^3^; Average			
		5.4 particles/m^3^			

polyamide (PA); polyacrylonitrile (PAN); polyethylene (PE); polyethylenimine (PEI); poly (ethylene phthalate) (PEP); polyethylene terephthalate (PET); polyethylene-vinyl-acetate (PEVA); poly (methyl acrylate) (PMA); polypropylene (PP); polystyrene (PS); polyester urethane (PU); poly (vinyl alcohol) (PVA); polyvinyl chloride (PVC); vinyl chloride/vinyl acetate copolymer (PVCA); poly (N-vinyl carbazole) (PVK); styrene butadiene rubber copolymer (SBR); extruded polystyrene (XPS).

### Dermal

Despite the number of personal care products such as scrubbing powder cleaners, shampoo, facial make-up, and balsams containing MPLs and microbeads in their formulations, and the fact that it has been suggested that there is a potential for NPLs to cross the dermal barrier (Revel et al., 2018), little attention has been paid to assessing dermal exposure. In one of the first studies to assess the potential cytotoxicity of MNPLs, Schirinzi et al. (2017) (Schirinzi et al., 2017) evaluated the effects on human epithelial cells of PE and PS, showing that PS presented Reactive oxygen species (ROS) generation. This is another indication of the importance of assessing the different plastic polymers, rather than considering MNPLs of different materials under the same umbrella. MPLs, and in particular NPLs due to their small size, 3D structure, and apolarity of the majority of polymers, can be internalised by cells (Xu et al., 2019). Moreover, the potential interaction between NPLs and proteins could lead to structural changes of proteins (Hollóczki and Gehrke, 2019) as well as the disruption of the lipid bilayer (Hollóczki and Gehrke, 2020).

## Pathways of Micro and Nanoplastics in Human Toxicity

MNPLs have a complex nature involving a polymeric matrix with additives such as plasticisers, flame retardants, fillers, UV stabilisers, coating finishers, colourants, metals, among others. Thus, firstly, MNPL toxicity should consider the physical particle damages that can cause inflammatory lesions, originating from the potential of their surface to interact with the tissues. Secondly, MNPL toxicity should also consider the long-term toxicity produced by the plastic additives, which are not covalently bound to the polymer and, once internalised, could be easily released.

Nowadays, most of the information about MNPL toxicity impacting human health is from a limited number of *in vitro* studies and relevant gaps of information exist. According to the information gathered to date, the Science Advice for Policy by European Academies (SAPEA) project in 2019 considered the lack of evidence on direct adverse effects of MPLs on human health (Sapea, 2019). Furthermore, considering the number of current uncertainties, the European Commission Scientific Advice Mechanism also considered the need to research this field in order to obtain a global perspective compared to other contaminants (not available in Crossref, 2018). As shown in Table 3, some effects cannot be overlooked, and some are dependent on the plastic particle composition, size, and shape. In order to assess the potential toxicity of MNPLs, the capacity of cell internalisation is a central issue. Liu et al. (2021a) (Liu et al., 2021a), using a model cell membrane and rat basophilic leukaemia (RBL-2H3) cells, evaluated the cellular internalisation and release of PS-MNPLs. In that study, both models were exposed to 50, 500, and 5,000 nm particles. Those authors showed that 50 and 500 nm particles were absorbed on the model membrane due to hydrophobic interactions and Van der Waals forces (meaning dispersion forces). This range of particles was also internalised into living cells via both 1) passive membrane penetration because of the partition of PS-MNPLs in the water-phospholipid system and 2) in the case of 50 nm particles by active endocytosis through the clathrin and caveolin-mediated pathways and mainly by micropinocytosis. As expected endocytosis opens the plasma membrane to Ca^2+^ influx which could cause the mitochondria dysfunction. In contrast, bigger particles (5 μm cannot be internalised due to their size. The endocytosed 50 and 500 nm particles mainly accumulate in the lysosomes. In addition to cell internalisation, these particles were also excreted. Finally, it was concluded that masses of the 50 nm particles that were internalised and excreted were both higher than the masses of 500 nm (Liu et al., 2021a).

**TABLE 3:** Selection of toxicological studies using human cell-lines.

**Cells**	**Cell-tissues**	**Particle characteristics**	**Effect evaluated**	**References**
RBL-2H3	Mast	PS-MNPLs of 50, 500, and 5,000 nm	Internalisation and release	Liu et al. (2021a)
Caco-2	Human colon	PS-MNPLs of 100 and 5,000 nm	Cytotoxicity (cell viability, oxidative stress, and membrane integrity).	Wu et al. (2019)
Caco-2	Human colon	PS-MNPLs of 100 and 5,000 nm	Cytotoxicity (cell viability and genomics).	Wu et al. (2020)
Caco-2	Human colon	Different compositions	Cytotoxicity (Inflammatory endpoints, including the cytokines IL-8, TNFα and IL-1β, as well as changes in the barrier integrity).	Lehner et al. (2020)
HT29-MTX-E12, human blood monocyte-derived macrophages and dendritic cells		50 and 500 µm		
Caco-2	Human colon	PS-MNPLs of 100 and 5,000 nm	Cytotoxicity comparison between digested and pristine particles	Liu et al. (2020)
		Pristine and transformed particles by digestive process		
Caco-2, HepG2 and HepaRG	Human colon and liver	PE, PP, PET and PVC	Uptake and transport	Stock et al. (2021)
		1–4 μm		
A549	Human lung alveolar epithelial cells	PS-NPLs	Internalisation, cell viability, cell cycle, apoptosis, and associated gene transcription and protein expression	Xu et al. (2019)
		25 and 70 nm		
BEAS-2B	Human lung normal epithelial cells	PS-MPLs with an average size of 1.72 ± 0.26 μm	Cytotoxic and inflammatory effects	Dong et al. (2020)
A549	Human lung alveolar epithelial cells	PS-MPLs) of 1 and 10 μm diameter	Cell proliferation, cytotoxicity	Goodman et al. (2021)
			Metabolic activity	
T98G and HeLa		PS-MNPLs	Cytotoxicity (cell viability and ROS effect)	Schirinzi et al. (2017)

Polystyrene (PS); polypropylene (PP); polyethylene terephthalate (PET); polyvinyl chloride (PVC).

Since ingestion is considered to be the first mode of human exposure, the gastrointestinal tract is one of the first exposure tissues. For this reason, several studies have been carried out to explore the responses in the human colon adenocarcinoma Caco-2 cells as a model. Wu et al. have studied whether cytotoxicity of PS particles was size-dependent in exposing Caco-2 cells to PS-MNPLs of 5 μm and 100 nm (Wu et al., 2019). In that study, both particle sizes exhibited low toxicity on cell viability, oxidative stress, and membrane integrity. However, the mitochondrial membrane potential was disrupted by both, and on the contrary, as expected, the 5 μm particles induced higher effects than those of 100 nm (Wu et al., 2019). In this case the authors explained that might be due to the different mechanisms of induced mitochondrial depolarization. It was found that 0.1 μm PS-MPs accumulated in lysosomes, while 5 μm PS-MPs analyses did not reveal accumulation in lysosomes. Large particles at a micrometer scale could escape from the lysosomes after endocytosis and localize in the intracytoplasmic vacuoles or randomly in the cell cytoplasm, which might further damage the lysosomes and induce mitochondrial depolarization and cell apoptosis. Thus, the results from different studies should be compared with caution. In another study by another group, Wu et al. (2020) investigated the cytotoxicity and the transcriptomic profiles of PS particles in human Caco-2 cells (Wu et al., 2020). That work demonstrated that PS-MPLs reduced cell viability in a dose-dependent manner. The responsible genes were identified by Illumina RNA seq. The dominant pathways related to NF-κB, MAPK signalling, cytokine-cytokine receptor interaction, and toll-like receptors, which are involved in modulating cell inflammation and proliferation were strongly influenced. Moreover, the qPCR was applied to investigate the transcriptional level of five proliferation-related genes (Ras, ERK, MER, CDK4, Cyclin D1) and four inflammation-related genes (TRPV1, iNOS, IL-1β, IL-8), and the results were consistent with RNA-seq data. Lehner et al. (2020) (Lehner et al., 2020) designed a novel, three-dimensional *in vitro* intestinal model, consisting of the human intestinal epithelial cell lines Caco-2 and HT29-MTX-E12 as well as human blood monocyte-derived macrophages and dendritic cells, that is suitable for assessing the possible effects of ingested MPLs of different composition in automobile tyre wear and polyolefins in a range of sizes between 50 and 500 µm. MPL particles were exposed at concentrations of 823.5–1,380.0 µg/cm^2^ directly on the intestinal model’s surface. Cytotoxicity was investigated after 6, 24 and 48 h of exposure by measuring the release of lactate dehydrogenase. Inflammatory endpoints, including the cytokines IL-8, TNFα and IL-1β, as well as changes of the barrier integrity after exposure, were additionally monitored. The main results of this study were that the MPLs between 50 and 500 µm did not cause any significant cytotoxicity or release of (pro-)inflammatory cytokines and did not change the barrier integrity the points investigated, at any of the time. In another interesting study (Liu et al., 2020), using PS-MPLs (100 and 5,000 nm), the influence of the digestive process on intestinal toxicity was also studied by using the *in vitro* Caco-2 model (Liu et al., 2020). The toxicity of original and transformed MPLs was studied. Results showed that the digestive process did not alter the chemical constitution but formed a corona on the surface of MPLs. The 100 nm PS-MPLs showed higher intestinal toxicity than 5 μm PS-MPLs. Digestive treatment relieved cytotoxicity and transport function disorder of the Caco-2 monolayer at was induced by non-treated PS-MPLs. However, the *in vitro* digestive process increased the proinflammatory effects of PS-MPLs. The formation of a corona on the surface leads to a change in size, Zeta potential, and adsorbed compounds. Also, in the same line, Wang et al. (2021a) (Wang et al., 2021a) investigated the cytotoxicity of transformed PS-MPLs (presenting alterations in the surface of the particles, chemical composition, and changes of the surface charge) in hepatocytes. The results of that study revealed that 500 nm PS-MPLs, which were chemically transformed by simulated gastric fluid, exacerbated their toxicity on SMMC-7721 cells at 20 μg/ml after 24 h of treatment. The observed effects included morphological alteration of cells, membrane damage and increased cell apoptosis via oxidative stress. The authors hypothesised that these effects could be partially explained by the degradation and surface modifications of PS-MPLs. In conclusion, transformed PS-MPLs presented higher hepatic cytotoxicity after transformation by simulated gastric fluid.

The GIT uptake and effects of different MPLs of most common polymers (PE, PP, PET and PVC) were investigated by Stock et al. (Stock et al., 2021) using the Caco-2 cell line as the *in vitro* cell-line model and using the cytotoxicity of the human cell lines Caco-2, HepG2 and HepaRG in order to detect a possible impact on the organs which are first to come into contact with ingested particles: the intestine and the liver. The results of the study demonstrate that especially 1–4 μm PE-MPLs were transported through the intestinal epithelium and that intestinal exposure to MPLs is material- and size-dependent. However, it should be highlighted that only a high concentration far beyond realistic dietary exposure of consumers induced cytotoxic effects.

Although much less investigation has been undertaken in exploring the potential cytotoxicity of MNPLs in other tissues, several studies were carried out during recent years. Xu et al. (2019) (Xu et al., 2019) carried out a preliminary evaluation of the effects of PS-NPLs on human lung epithelial cells. The internalisation, cell viability, cell cycle, apoptosis, and associated gene transcription and protein expression were assessed on the human alveolar epithelial A549 cell line exposed to 25 and 70 nm PS-NPLs. Results showed that smaller particles were internalised more rapidly and efficiently into the cytoplasm of A549. PS-NPLs significantly affected the cell viability, induced significant up-regulation of pro-inflammatory cytokines such as IL-8, NF-κB, TNF-α, and pro-apoptotic proteins such as DR5, caspase-3, caspase-8, caspase-9, and cytochrome c, which revealed that it triggered a TNF-α-associated apoptosis pathway. That study suggests that exposure duration, diameter, and concentration are the key factors in the toxicological effects of PS-NPLs on alveolar epithelial cells. In another study by Dong et al. (2020) (Dong et al., 2020), the potential of *in vitro* pulmonary toxicity of PS-MPLs was assessed using normal human lung epithelial BEAS-2B cells. PS-MPLs with an average size of 1.72 ± 0.26 μm were used. Results revealed that PS-MPLs could cause cytotoxic and inflammatory effects in BEAS-2B cells by inducing reactive oxygen species (ROS) formation. PS-MPLs can decrease transepithelial electrical resistance by depleting zonula occludens (ZO) proteins. According to the authors, high concentrations of PS-MPLs produce the decreased α1-antitrypsin levels, increasing the risk of chronic obstructive pulmonary disease. Furthermore, low levels of PS-MPLs can disrupt the protective pulmonary barrier, and they may also increase the risk of lung disease. These findings indicate that PS-MPL inhalation may negatively influence human respiratory health. The potential toxicological damages of PS-MPLs in lung cell were also proposed by Goodman et al. (2021) (Goodman et al., 2021). In that study, human alveolar A549 cells were exposed to polystyrene microplastics (PS-MPLs) of 1 and 10 μm diameter. Both sizes caused a significant reduction in cell proliferation but exhibited little cytotoxicity. Despite these results, further assays revealed the metabolic activity decrease and a dramatic decrease in the proliferation rate of exposed cells. Phase-contrast imaging of live cells revealed at 72 h major changes in exposed cells morphology, as well as the uptake of 1 μm particles into the cells. Confocal fluorescent microscopy at 24 h of exposure confirmed the incorporation of 1 μm PS-MPLs.

Other studies have focussed on the potential genotoxic effects of PE on peripheral blood lymphocytes (Çobanoğlu et al., 2021) and the ROS effect on T98G and HeLa, cerebral and epithelial human cells, respectively (Schirinzi et al., 2017). In conclusion, the main toxicological effects that can be attributed to MNPLs particles are oxidative stress, cytotoxicity, and metabolic changes. However, the composition of the particles and the dose drive the toxicological properties, size, shape, surface functionalisation, or surface properties.

## Pathways of Plastic Additive Toxicity

Plastic formulations are composed of polymer or polymer mixtures and plastic additives, including functionalising agents (plasticisers, impact modifiers, and flame retardants), stabilisers (antioxidants, UV-filters, thermal stabilisers), lubricants, fillers, and colourants. The additives are incorporated into the polymers during manufacture, but they are not chemically bound to them, as aforementioned. Due to this reason and their low molecular weight, plastic additives can leach the external medium along a concentration gradient (Tickner et al., 2001).

As mentioned before, when particle size decreases, the surface-active area in contact with the external medium will increase, facilitating the leach. If MNPLs accumulate in living organisms, they present a source of chemical plastic additives and polymer monomers to tissues and fluids.

Indeed, the impacts on human health of some selected groups of plastic additives such as polybrominated diphenyl ethers (PBDEs) (Maddela et al., 2020), bisphenol A (Kahn et al., 2020; Meli et al., 2020; Mustieles and Fernández, 2020a), and some phthalates (Sedha et al., 2021) have been extensively studied in the last 10 years, and relevant hazard and epidemiological information (Husøy et al., 2019) have been collected. These chemicals have been related to carcinogenicity, neurotoxicity (Mustieles and Fernández, 2020b; Li et al., 2020), obesity (Andújar et al., 2019), and endocrine disruption (Ma et al., 2019). Human studies showed that the concentrations of these additives in young children, a susceptible segment (Ma et al., 2019; Ouyang et al., 2020), are typically higher, indicating the need to decrease exposure to these compounds. Thus, a wide variety of substitution compounds have arisen in new plastic formulations, including emerging plasticisers, brominated flame retardants (BFR) and organophosphate esters (OPEs) in substituting PBDE legacies, or those used in new plastic materials such as bioplastics. Bioplastics derived from renewable biomass sources such as polylactic acid (PLA), polyhydroxyalkanoates (PHA), bio-derived polyethylene (Bio-PE), among many others, offer a greener solution for plastic production. However, very little information is currently available on their impact on living organisms, including humans. In this sense, Zimmermann et al. (2019) (Zimmermann et al., 2019) demonstrated that consumer products made of PLA induced strong baseline toxicity similar to PVC items. This demonstrates that this bio-based and biodegradable material, despite being marketed as better alternative, is not necessarily safer than conventional plastics. Despite the amount of research that has been devoted to assessing the potential impact of certain groups of plastic additives, such as some plasticisers and flame retardants, the information about the other groups continues to be scant. As can be seen in Table 4, this tendency has continued in recent years and most of the investigations have been focussed on plasticisers of the group of phthalates and bisphenols, and the new organ phosphate flame retardants. In addition to many toxicological studies showing the negative effects of certain phthalate congeners on human health, such as asthma, breast cancer, obesity, type II diabetes, and male infertility, the potential health damages by new substitution compounds, the phthalate transformation products, or even human metabolites of primary phthalates are open questions. In this regard, Sicińska (Sicińska, 2018), undertook an *in vitro* study of the induction of haemolysis and eryptosis in human erythrocytes caused by di-n-butyl phthalate (DBP), butyl benzyl phthalate (BBP), and their metabolites: mono-n-butyl phthalate (MBP) and mono-benzyl phthalate (MBzP). The main results of this study demonstrated that DBP and BBP possess higher haemolytic properties compared to their metabolites, but all compounds induced eryptosis.

**TABLE 4:** Examples toxicological assays of plastic additives using human cell-lines.

**Additives class**	**Chemical compounds**	**Test**	**Effect**	**Results summary**	**Ref**
Plasticisers	Adipate: DEHA Phtalates: DEHP, DBP	Steroid hormone synthesis in H295 R cells	Endocrine disruption. Effects on steroid hormone synthesis	Even at low concentrations of exposure (≤1 mg/L or 2.5 μM) these chemicals and their metabolites can cause significant endocrine disrupting effects	Duan et al. (2020)
	Bisphenols: BPA, BPF, BPS, TBBPA	Human embryonic stem cells H9 using RNA-sequencing exploring impacts in the estrogen receptor negative	Effects of BPA and its analogues in stem cells to explore potential developmental impacts	BPA, BPF, and BPS have similar potencies in inducing transcriptional changes and perturb many of the same pathways. TBBPA, the least structurally similar of the group, exhibited much lower disrupting potency	Peshdary et al. (2021)
	Bisphenols: BPA, NP, OP	Human placenta JEG-3 cells	Cytotoxicity	After 24 h exposure, OP and NP showed the highest cytotoxicity (EC_50_: 36–40 μM) followed by BPA (138–219 μM), whereas no significant toxicity was observed for phthalates. Notwithstanding, BBP and DBP significantly decreased P450 aromatase activity, while NP and OP increased the activity	Pérez-Albaladejo et al. (2017)
	Phthalates: (BBP), (DBP), (DEHP), (DMP)		ROS effect		
	DINCH	human liver and kidney cell lines	cytotoxicity and genotoxicity	DINCH produced oxidative DNA transient damage in liver cells exposed for 3 h. DINCH may be hazardous to humans and further investigation is necessary to warrant its safety	Vasconcelos et al. (2019)
	Phtalates: DEHP	Human embryonic stem cells	Endocrine disruption and embriotoxicity	Inhibition of cell proliferation, promotion of cell cycle arrest, and induced apoptosis through the PPARγ/PTEN/AKT signalling pathway. Suggesting potential reproductive or developmental toxicity	Fang et al. (2019a)
		H9-hESC			
	Phtalates: DEHT and their human metabolites 5-OH-MEHP and MEHT	Thyroid/hormone receptors	Endocrine disruption	lack of interactions between oxidised metabolites and thyroid hormone receptors, confirming the interest in DEHT as substitute of DEHP	Kambia et al. (2021)
	Phthalates and PE-NPLs: BBP, DBP, DEHP	Human lung epithelial A549 cells	Cytotoxicity (oxidative stress and inflammation)	Oxidative stress and inflammatory reactions were mechanisms for combined cytotoxicity	Shi et al. (2021)
	Phthalates: BBP, DBP, DEHP	Human keratinocyte cell line HaCaT	Cytotoxicity	ATEC showed similar levels of cytotoxicity with the phthalates, whereas ATBC and ATHC did not show significant cytotoxicity. even in high doses (5 mg/ml)	Kim et al. (2019)
	Phtalate substitutes: ATBC, ATEC, ATHC				
	Phthalates: DBP, BBP, and their metabolites: MBP and MBzP	Human erythrocytes cytotoxicity	Cytotoxicity (haemolysis and erytopsis)	DBP and BBP possess higher haemolytic properties compared to their metabolites	Sicińska, (2018)
				LC_50_ was 126.37 ± 5.94 μg/ml for DBP, and 103.65 ± 4.03 μg/ml for BBP, and for metabolites this value was over 500 μg/ml	
	Phthalates: DEHP and MEHP	Alveolar epithelial A549 cells	Evaluation of the cell progression, epithelial and mesenchymal markers	DEHP and MEHP altered the structure and migration of A549 cells and promoted the loss of the epithelial phenotype	Rafael-Vázquez et al. (2018)
	Flame retardants	Organophosphate	Human liver hepatocellular carcinoma cell line, HepG2	changes in gene transcription	TBOEP treatments resulted in increases in cell metabolism could explain the increase in mitochondrial activity at lower TBOEP concentrations. In addition, showed effects on steroid hormone biosynthesis and regulation, and potentiation of immune responses	Krivoshiev et al. (2018)
		TBOEP				
		Organophosphate	Human liver hepatocellular carcinoma cell line, HepG2	Hepatotoxicity	HepG2 exposed to the highest concentration of TCP (400 μM) for 3 days showed 49.85% decline in survival, DNA damage in cells, dysfunction of mitochondrial membrane potential. The cell cycle analysis exhibited 62.53% cells in the subG1 apoptotic phase. qPCR array of 84 genes unravel the transcriptomic alterations in HepG2 cells. These effects confirm the hepatotoxic potential of TCP.	Al-Salem et al. (2019)
		TCP				
		Organophosphate: EHDPP, TPCP, TOCP, TPHP, TCP and CDP	Human normal liver hepatocytes (L02)	Hepatotoxicity	10 mg/L of EHDPP significantly affected energy homeo-stasis, endoplasmic reticulum (ER) stress, apoptosis, cell cycle, and inflammation response in cells	Zhu et al. (2021)
		PBDEs: quinone-type metabolite of PBDEs (PBDEQ)	Human normal liver hepatocytes (L02)	Hepatotoxicity	PBDEQ-induced protein oxidative damage in LO2 cells	Wang et al. (2021b)

tributyl o-acetylcitrate (ATBC); triethyl 2-acetylcitrate (ATEC); trihexyl o-acetylcitrate (ATHC); butyl benzyl phthalate (BBP); bisphenol A (BPA); bisphenol F (BPF); bisphenol S (BPS); diphenyl phosphate (CDP); di-n-butyl phthalate (DBP); bis(2-ethylhexyl) adipate (DEHA); bis(2-ethylhexyl) phthalate (DEHP); diisononyl cyclohexane-1,2-dicarboxylate (DICH); dimethyl phthalate (DMP); mono-n-butyl phthalate (MBP); mono-benzyl phthalate (MBzP); monoethylhexyl phthalate (MEHP); Nonylphenol (NP); octylphenol (OP); polybrominated diphenyl ethers (PBDEs); tetrabromobisphenol A (TBBPA); tris(2-butoxyethyl)phosphate (TBOEP); tricresyl phosphate (TCP); tri-o-cresyl phosphate (TOCP); tri-p-cresyl phosphate (TPCP); triphenyl phosphate (TPHP).

One of the most commonly used plasticisers is di (2-ethyl hexyl) phthalate (DEHP), which is metabolised to mono (2-ethylhexyl) phthalate (MEHP), and inhalation is an important exposure route for both. Their effects on the lungs include inflammation and alteration of postnatal maturation (alveolarisation). Recently, the cell progression, epithelial and mesenchymal markers were studied in alveolar epithelial cells A549, exposed to DEHP (1–100 μM) or MEHP (1–50 μM) for 24–72 h (Sedha et al., 2021). That experimental work showed that DEHP and MEHP altered the structure and migration of A549 cells and promoted the loss of the epithelial phenotype (Rafael-Vázquez et al., 2018). DEHP has been classified as an endocrine disruptor. Fang et al. (Fang et al., 2019a) investigated the mechanism of the embryotoxicity induced by DEHP in differentiated human embryonic stem cells (hESCs). It was demonstrated that DEHP exposure inhibited cell proliferation, promoted cell cycle arrest, and induced apoptosis through the PPARγ/PTEN/AKT signalling pathway, suggesting that DEHP exposure could cause reproductive or developmental toxicity in humans (Fang et al., 2019a).

Due to the endocrine disruption effects of different phthalates, currently, a series of substitutes have been proposed, such as tributyl o-acetylcitrate (ATBC), triethyl 2-acetylcitrate (ATEC), and trihexyl o-acetylcitrate (ATHC). To assess the safety of these new substitutes, the potential cytotoxicity of these compounds was screened in a human keratinocyte cell line HaCaT. The results demonstrated that ATEC showed similar levels of cytotoxicity as common phthalates, whereas ATBC and ATHC did not show significant cytotoxicity, even at very high doses (5 mg/ml) (Kim et al., 2019).

Another plasticiser with high usage in the past in polycarbonate plastic production is bisphenol-A (BPA). However, BPA exhibits toxicity, endocrine disruption, mutagenicity and carcinogenic effects in living organisms (Maffini et al., 2006; Rochester, 2013). For these reasons, in 2011, the plasticiser use was banned in baby bottles, cups, and other containers that are designed for use by children of 3 years old and younger, and in 2012 their use was also banned in water bottles. Currently, the toxicity of other alternative compounds of this group, such as bisphenol-F (BPF) and bisphenol-S (BPS), and 3,3′,5,5′-tetrabromobisphenol-A (TBBPA) that are also used as a flame retardant, are being investigated. Further, several studies have revealed that these compounds also presented toxicity in specific BPF and BPS (Fouyet et al., 2021).

Another group of plasticisers with increasing use are the adipates, this class of plasticisers has been much less studied but recent findings showed that bis (2-ethylhexyl) adipate (DEHA) and their metabolites can cause, even at low concentrations, endocrine-disrupting effects on the steroid hormone synthesis in H295R cell (Duan et al., 2020).

As aforementioned, flame retardants are another group of plastic additives that has been related to different toxicological effects in human health. In particular, some PBDEs are currently listed in Annex A of the Stockholm Convention on Persistent Organic Pollutants with specific exemptions for use regarding the recycling of articles that contain or may contain these chemicals. Thus, these compounds have been replaced by other groups, among them organophosphate-flame retardants. Currently, there are some gaps of information about their fate, behaviour and toxicity, but some compounds have been related to human accumulation (Chupeau et al., 2020) and health impairments (Wang et al., 2020a; Chupeau et al., 2020). Among them, there are five main toxicological effects described as reproductive and developmental toxicity (Liu et al., 2021b), neurotoxicity (Yan et al., 2021), hepatotoxicity (Zhu et al., 2021), cardiotoxicity (Reddam et al., 2019; Xiong et al., 2021) and endocrine disruption (Liu et al., 2019a). However, most of these studies were *in vivo* assays with animals, and some information of toxicity mechanisms from *in vitro* tests using human cell is still required.

Other chemicals that could leach from the plastic polymer matrix include antioxidants, UV stabilisers such as the group of benzophenones and plastic coating finishing compound such as perfluoroalkyl substances (PFASs) (Pelch et al., 2019), but with the exception of the PFASs group, the potential for adverse effects on human health of other plastic additives has been much less studied.

It is noteworthy that, currently, more than 5,300 plastic formulations are commercially available. More than 4,000 known chemicals are associated with plastic packaging alone, and to date most of these chemicals have not been appropriately studied.

Moreover, in general, the potential migration of additives from the plastics in use is the considered factor for the evaluation of their potential impact. In the case of MNPL contamination from different sources, the potential migration from MNPLs particles during long-term exposure should be assessed. Lixiviation should be evaluated from particles of the smallest range of sizes, with modified surface properties, considering the potential accumulation in tissues, and sometimes coming from plastic formulations that are no longer in use, with residues that are still in the environment.

## Trojan-Horse Effects and Microbiome Effects

The increased surface area of MNPLs and their surface properties, such as hydrophobicity and adsorption, favour the accumulation onto their surfaces of other contaminants from surrounding environments, facilitating their potential transfer to living organisms, including humans. Llorca et al. (2018) (Llorca et al., 2018) assessed the adsorption and desorption behaviour of 18 perfluoroalkyl substances (PFASs) from the surrounding waters (freshwater and seawater) of Spain onto MNPLs surfaces of different polymers, including PE, PS, and polystyrene carboxylate (PS-COOH)). Then, the same group investigated the capacity for the transfer of polychlorinated biphenyls (PCBs), a group of persistent organic pollutants (POPs) to PE, PS, and PET MPL surfaces in water/sediment systems (Llorca et al., 2020b). In both studies, the adsorption/desorption of other contaminants onto MNPL surfaces was confirmed, mainly following the Freundlich isotherms. Medium polar compounds showed that the adsorption and posterior desorption increased. While highly non-polar compounds were sequestered onto the MNPL surfaces. Therefore, it should be considered how the interaction of contaminant/MPLs can affect the absolute bioavailability of these contaminants that are transferred to biota. Indeed, several works informed about the potential for the accumulation of environmental pollutants onto MPL surfaces (Lang et al., 2020; Atugoda et al., 2021; Fan et al., 2021; Puckowski et al., 2021; Torres et al., 2021). However, less research has been paid to assessing of whether certain tissue temperatures and physiological conditions can increase the potential transfer of other contaminants to humans. In this regard, the potential for POPs (DDT, PFOA, and DEHP) to desorb from MPLs of PVC and PE under simulated physiological conditions has been studied (Bakir et al., 2014). Desorption rates were enhanced up to 30 times compared to seawater at lower pH and higher temperatures, in conditions simulating the physiological conditions of warm organisms, 38°C, pH 4. Therefore, this pathway of human exposure needs to be addressed. In general, the toxicological interaction between MNPLs and other contaminants has been assessed using *in vivo* studies, the majority of which were focused on the effect on model organisms exposed to a mixture of chemicals or an individual chemical but in both cases at a single concentration. Therefore, dose-response curves were not provided, and a complete description of these interactions was not assessed.

Several factors rule the modulation of the toxicity of MNPLs and other contaminant toxicity such as physicochemical properties of MNPLs and contaminant mixtures, and therefore, their adsorption/desorption behaviour, and that of the selected model organisms. Other essential factors are the size and shape of MNPLs, and even with respect to the colour for some test organisms (Bhagat et al., 2021).

On the other hand, MNPL surfaces are colonised by microbes. In the natural environment, for example, biofilms can be established on plastic particle surfaces within a period of 7 days. Thus, the potential transfer of pathogens via microplastics that are inhaled or ingested should also be investigated.

Finally, another potential effect that should be closely studied is the MNPLs potential to disrupt the human microbiome. The chronic ingestion of MNPLs could impact on the natural community and abundance pattern of the gut microbiota. This so-called dysbiosis could be associated to mechanical disruption within the GIT, the ingestion of foreign and potentially pathogenic bacteria, as well as chemicals, which make-up or adhere to MNPLs. The impact on the microbiome can result in the immune system response and trigger chronic diseases, promote infections, and alter the gut microbiota. For example, lipopolysaccharides (LPS) are able to adjust the hydrophobicity in their cell walls by changing their composition in the outer membrane, to better interact with other hydrophobic substances (Krasowska and Sigler, 2014; Rogers et al., 2020). LPS also can effect gut microbiota so changes on LPS will direct affect microbiota (Di Lorenzo et al., 2019). In this regard, Fackelmann and Sommer (Fackelmann and Sommer, 2019) have summarised microplastics-induced gut dysbiosis effects on host health, and some few pioneering studies have been carried out during the last years, studying responses on mice (Lu et al., 2018) and aquatic organisms (Evariste et al., 2019), but this is another human open question for human health implications.

## Current Needs and Future Research Trends

The study of the potential impacts of MNPLs in human health is in an early stage of development. Firstly, the analytical approaches to assess them are limited by the size of the particles, and there are only a few approaches able to assess the particles at the nano-range. Thus, human exposure assessment continues to be a challenge. Moreover, it is expected that small-sized particles are those with a superior rate of up-take and translocation through tissues, but this concordance is not always observed, and it should be better studied. On the other hand, the interaction of MNPLs to lipopolysaccharides needs to be further investigated since the effects of LPS on the gut plays an important role and their release into the blood will damage the endothelium. This needs to be examined because the effects of LPS on the gut is important and the release of LPS into the blood will damage the endothelium.

Particle weathering is also a parameter that should be considered when assessing toxicity of MNPLs- Physical damages caused by particles include the disruption of the immune system, the increase of barriers permeability, leading to oxidative stress and inflammation, favouring the uptake of smallest particles and translocation through tissues. In addition, their higher surface area favours the long-term leach of plastic additives or the transfer to the biota of other contaminants and maybe pathogens. Besides, the potential disturbance of the human microbiome can be another cause of human health damage almost not studied until now.

The main routes of human exposure have already been established, with ingestion and inhalation being the main routes. Nevertheless, the occurrence of MNPLs in complete diets has not been established to date. Therefore, the total contribution of MNPLs to human exposure of exogenous particles through ingestion is still unknown. Moreover, incidental sources of exposure (MNPLs from food packaging, ingestion from PPCs, among others, have not been well determined). Inhalation is another relevant source of MNPL exposure, but relatively few studies have considered indoor exposure.

There is a need for more comprehensive toxicological studies, including particulate physical damages and the toxicity of additives. In this regard, the information about plastic additive toxicity continues being limited to targeted groups of compounds such as phthalates. In addition, the toxicity of MNPLs is also modulated by their size, shape, and studies comparing these variables are necessary.

Finally, to assess the impacts of MNPLs contamination and their potential damages to human health, cohort studies focussed on this emerging type of contamination are required.

## Data Availability

The original contributions presented in the study are included in the article/Supplementary Material, further inquiries can be directed to the corresponding authors.
